# Socially Assistive Robots for Pain Management and Emotional Responses in Pediatric Hospital Care: Systematic Review and Meta-Analysis

**DOI:** 10.2196/76427

**Published:** 2025-11-26

**Authors:** Fang Yu Hsu, Yun Hsuan Lee, Jia-Ling Tsai, Angela Shin-Yu Lien

**Affiliations:** 1 Department of Nursing Linkou Chang Gung Memorial Hospital Taoyuan Taiwan; 2 Graduate Institude of Nursing College of Medicine Chang Gung University Taoyuan Taiwan; 3 School of Nursing College of Medicine Chang Gung University Taoyuan Taiwan; 4 Department of Endocrinology and Metabolism Linkou Chang Gung Memorial Hospital Taoyuan Taiwan

**Keywords:** socially assistive robots, child, hospitalization, pain, anxiety, fear, distress, systematic review, meta-analysis

## Abstract

**Background:**

Pain and emotional distress are prevalent concerns in pediatric hospital care, underscoring the need for safe and evidence-based nonpharmacological interventions. Socially assistive robots (SARs) are innovative tools that alleviate pain and emotional distress through social interaction. Although previous reviews suggest potential benefits, the evidence remains ambiguous, with insufficient exploration of the contextual factors influencing the effective implementation.

**Objective:**

This systematic review with meta-analysis evaluates the effectiveness of SARs in reducing pain and emotional outcomes in pediatric hospital settings and identifies methodological, contextual, and ethical factors informing future implementation.

**Methods:**

Following the PRISMA (Preferred Reporting Items for Systematic Reviews and Meta-Analyses) 2020, 8 databases (PubMed, MEDLINE, Embase, Cochrane Library, Scopus, IEEE Xplore, Health & Medical Collection, and ProQuest Dissertations & Theses A&I) were searched until October 7, 2025. Eligible studies were randomized controlled trials involving participants aged <19 years in hospital settings. Two reviewers independently screened, extracted data, assessed risk of bias, and evaluated the certainty of evidence. Random-effects meta-analyses were performed using the Hartung-Knapp-Sidik-Jonkman method. Both CIs and prediction intervals (PI) were interpreted.

**Results:**

Thirteen randomized controlled trials were included, of which 7 were eligible for meta-analyses. The narrative synthesis, incorporating intervention characteristics and contextual factors, suggested potential psychological benefits. For pain, a pooled analysis of 5 trials showed a significant reduction favoring SARs (difference in means=–0.89, 95% CI –1.32 to –0.47; 95% PI –1.29 to –0.49; *I*²=11.9%, τ²<0.0001, τ<0.01). For anxiety (3 trials; difference in means=–1.00, 95% CI –2.44 to 0.44; 95% PI –3.45 to 1.45; *I*²=73.8%, τ²=0.217, τ=0.466), fear (2 trials; difference in means=–0.04, 95% CI –1.72 to 1.64; *I*²=0%, τ²=0), and distress (2 trials; difference in means=–0.23, 95% CI –6.00 to 5.54; *I*²=65%, τ²=0.269, τ=0.519), pooled effects were nonsignificant with wide PIs. The GRADE (Grading of Recommendations, Assessment, Development, and Evaluation) assessment indicated an overall moderate certainty of evidence, limited primarily by the risk of bias, due to the nonblinded nature of the interventions and the small number of studies.

**Conclusions:**

SARs may serve as promising nonpharmacological adjuncts for pediatric pain management, demonstrating consistent and reproducible benefits across similar hospital contexts. In contrast, the evidence for emotional outcomes remains ambiguous. The PI indicating that while some children may experience emotional benefits, others may show null or even opposite effects. This variability highlights the real-world implications for the clinical implementation of SARs. Moreover, this study offers an innovative integration of contextual, ethical, and technological perspectives into a comprehensive synthesis, providing a multidimensional understanding of how SARs function within pediatric health care environments. Future research should adopt rigorous designs and incorporate ethical considerations to optimize effects and ensure sustainable implementation of SARs in pediatric health care.

**Trial Registration:**

PROSPERO CRD420251026751; https://www.crd.york.ac.uk/PROSPERO/view/CRD420251026751

## Introduction

Pain is defined as “an unpleasant sensory and emotional experience associated with, or resembling actual or potential tissue damage” [1]. In pediatric health care, pain is one of the most frequently reported concerns, and when inadequately managed, it may lead to long-term physical, psychological, and developmental consequences [2,3]. These risks underscore the urgent need for effective and safe pain management strategies tailored for children.

Current clinical recommendations emphasize multimodal approaches that integrate both pharmacological and nonpharmacological strategies to optimize outcomes in the pediatric population [3,4]. Pharmacologically, ibuprofen is the most extensively studied nonsteroidal anti-inflammatory drug and is widely recognized for its efficacy and safety in acute pediatric pain [2]. However, best practice not only achieves effective analgesia but also aims to minimize risks by reducing overreliance on pharmacological interventions and incorporating evidence-based nonpharmacological approaches [5,6].

In this context, socially assistive robots (SARs) have emerged as a promising nonpharmacological intervention for alleviating pain and mitigating emotional distress in pediatric health care settings [7-9]. Through features such as embodiment, personalization, empathy, and attentional distraction, SARs provide emotionally supportive interactions without requiring physical contact [10]. Evidence indicates that SARs can reduce procedural pain, anxiety, and distress while promoting positive affect and supporting postoperative recovery [11-14].

This potential is particularly relevant in hospital environments, where children frequently undergo painful and distressing medical procedures, such as injections, blood draws, surgeries, and cancer treatments [15-18]. Inadequately managed pain and distress in these settings may contribute to delayed recovery, prolonged hospitalization, long-term psychological sequelae, and reduced treatment adherence [19]. Compared with outpatients, hospitalized children are more often exposed to repeated and invasive procedures, making effective emotional support and pain management especially critical [18].

Despite the growing interest, most existing systematic reviews of SARs have focused on outpatient applications, particularly in mental health or short-term procedural contexts, such as vaccinations and dental visits [8,10,20,21]. A few meta-analyses have examined SARs in clinical settings for outcomes such as anxiety [22], pain and negative affect during needle-based interventions [23], and psychological well-being [24]. Emotional responses are inherently subjective experiences [25,26]. However, previous meta-analyses included a blend of observer-rated and self-reported outcome measures. This study prioritized children’s self-reports, which are more accurately captured through their own perspective.

Furthermore, research on human-robot interaction highlights that the clinical implementation of SARs requires careful consideration of ethical dimensions, such as safety, privacy, and autonomy [27,28]. Ethical concerns also include children’s potential emotional overdependence, unintentional attachment, and reduced meaningful human interaction, which are especially salient for younger patients undergoing emotional and social development [27,29]. However, these dimensions have received limited systematic attention in pediatric care.

To address these gaps, this systematic review with meta-analysis synthesizes findings exclusively from randomized controlled trials (RCTs) that evaluated the effectiveness of SARs in reducing pain and emotional outcomes, including anxiety, fear, and distress, among pediatric patients in hospital settings. In addition, this study provides a comprehensive synthesis of intervention design and contextual factors for future RCTs, ultimately improving clinical outcomes and enhancing children’s hospital experiences.

## Methods

### Study Design

This review was prospectively registered in the PROSPERO (International Prospective Register of Systematic Reviews; CRD420251026751). This study followed the PRISMA (Preferred Reporting Items for Systematic Reviews and Meta-Analyses) 2020 guidelines [30] and the PRISMA-S (Preferred Reporting Items for Systematic Reviews and Meta-Analyses Literature Search Extension) extension for literature searches (checklist provided in Multimedia Appendix 1) [31]. The search strategy was peer reviewed by a senior medical librarian before execution using the PRESS (Peer Review of Electronic Search Strategies) guidelines to ensure transparency, reproducibility, and methodological rigor [32]. Two reviewers independently conducted the study selection, risk of bias assessment, certainty of evidence appraisal, and data extraction. Discrepancies were resolved through discussions with a third reviewer and the corresponding author.

### Eligibility Criteria

This review included RCTs that met the following eligibility criteria according to the PICO framework: (1) population (P): participants were children <19 years of age in hospital settings; studies focusing on children diagnosed with autism spectrum disorder were excluded, as previous research has already established the efficacy of SARs in this population [33]; (2) intervention (I): involved the use of SARs, excluding studies focused on rehabilitation, training, or surgical applications; (3) comparison (C): studies included control or alternative intervention; and (4) outcomes (O): the primary outcome was pain. Secondary outcomes were emotion-related responses.

### Information Sources

A total of 8 electronic databases across 5 platforms were searched to identify relevant studies: PubMed (National Library of Medicine), MEDLINE (National Library of Medicine), Embase (Elsevier), Cochrane Library (Wiley), Scopus (Elsevier), IEEE Xplore Digital Library (IEEE Xplore), Health & Medical Collection (ProQuest), and ProQuest Dissertations & Theses A&I (ProQuest). To identify additional gray literature and unpublished studies, we searched the study registry ClinicalTrials.gov and manually screened conference proceedings from the Proceedings of the 2025 ACM/IEEE International Conference on Human-Robot Interaction. Both cited and citing references of relevant systematic reviews were examined by browsing their reference lists and using Google Scholar’s (Google LLC) citation function to identify additional eligible studies.

### Search Strategy

An iterative search strategy was developed following the PRISMA-S extension for the transparent and reproducible reporting of literature searches. The strategy combined Medical Subject Headings, related terms, and free-text keywords using Boolean operators to optimize the sensitivity and specificity. Search concepts were informed by the PICO framework and included terms related to “hospitalization,” “child,” “social robot,” “pain,” “distress,” “emotion,” “anxiety,” “fear,” and “well-being.” The search syntax was subsequently adapted to each database’s indexing system. The initial search was conducted on May 6, 2025, and updated on October 7, 2025, by rerunning the searches. No language or publication date restrictions were applied. The details of the search strategies, including full line by line search strings, filters, parameters, search dates, and retrieval counts, are presented in Multimedia Appendix 2.

### Selection Process

All references were imported into EndNote (version 21; Clarivate), and the duplicates were automatically removed. Titles and abstracts were independently screened by 2 reviewers, followed by full-text assessments based on predefined eligibility criteria. The reasons for exclusion are documented in Multimedia Appendix 2. The overall selection process is illustrated in the PRISMA flow diagram in the Results section.

A total of 1229 records were retrieved from 8 databases and 1 from citation searching. After removing 216 duplicates and screening titles or abstracts, 80 full texts were assessed. After 67 were excluded due to not meeting the criteria, 13 studies were included, with 7 providing sufficient data for meta-analysis.

### Quality Assessment

The methodological quality of the included RCTs was evaluated using the short version of the revised Cochrane Risk of Bias tool for randomized trials [34]. The risk of bias was assessed across 5 domains: randomization process, deviations from intended interventions, missing outcome data, outcome measurement, and selection of reported results. Each domain was rated as “low risk,” “some concerns,” or “high risk” of bias, and an overall judgment was made.

### Certainty of Evidence

The certainty of evidence for each outcome was assessed using the GRADE (Grading of Recommendations, Assessment, Development, and Evaluation) approach [35]. Five domains were evaluated: risk of bias, inconsistency, indirectness, imprecision, and publication bias. Outcomes were rated as “high,” “moderate,” “low,” or “very low” certainty of evidence. The ratings were generated using the GRADEpro Guideline Development Tool [36].

### Data Extraction and Synthesis

The data extraction included study characteristics such as authors, year of publication, country, study objectives, sample size, study population, participant age, setting, type of SARs, intervention details, comparator, measurement tools, and main findings. All the included studies contributed to the narrative synthesis. For the meta-analysis, only studies that provided sufficient numerical data were eligible for pooling, regardless of whether the outcome was primary (pain) or secondary (emotional responses). Where such data (eg, means, SDs, and sample sizes) were incomplete, we attempted to contact the original study authors to obtain additional information. Data synthesis was conducted in two parts: (1) narrative synthesis, summarizing key characteristics and findings of all included studies; and (2) meta-analysis, performed for outcomes with adequate quantitative data.

### Data Analysis

Meta-analyses were conducted using R version 4.2.1 (R Project for Statistical Computing). Pooled effect sizes were estimated using a random-effects model to account for anticipated heterogeneity [37]. The outcomes included pain, anxiety, distress, and fear. For each outcome, differences in means with corresponding 95% CIs were calculated to accommodate variability across measurement scales. Subgroup analyses or meta-regression were planned in the presence of substantial heterogeneity. Given the limited number of studies, the Hartung-Knapp-Sidik-Jonkman method was applied to adjust the SEs [38]. Between-study heterogeneity was quantified using the inconsistency index (*I*²), between-study variance (τ²) and SD (τ), and 95% prediction intervals (PI) were reported to indicate the expected range of effects in future studies, except for outcomes with very few studies [39]. Forest plots were generated to visualize the pooled effect sizes. Funnel plots were constructed to assess the small-study effect. As recommended, Egger test was not performed for outcomes with fewer than 10 studies because of its low statistical power to detect true asymmetry [40,41].

## Results

### Literature Search

**Figure 1:**
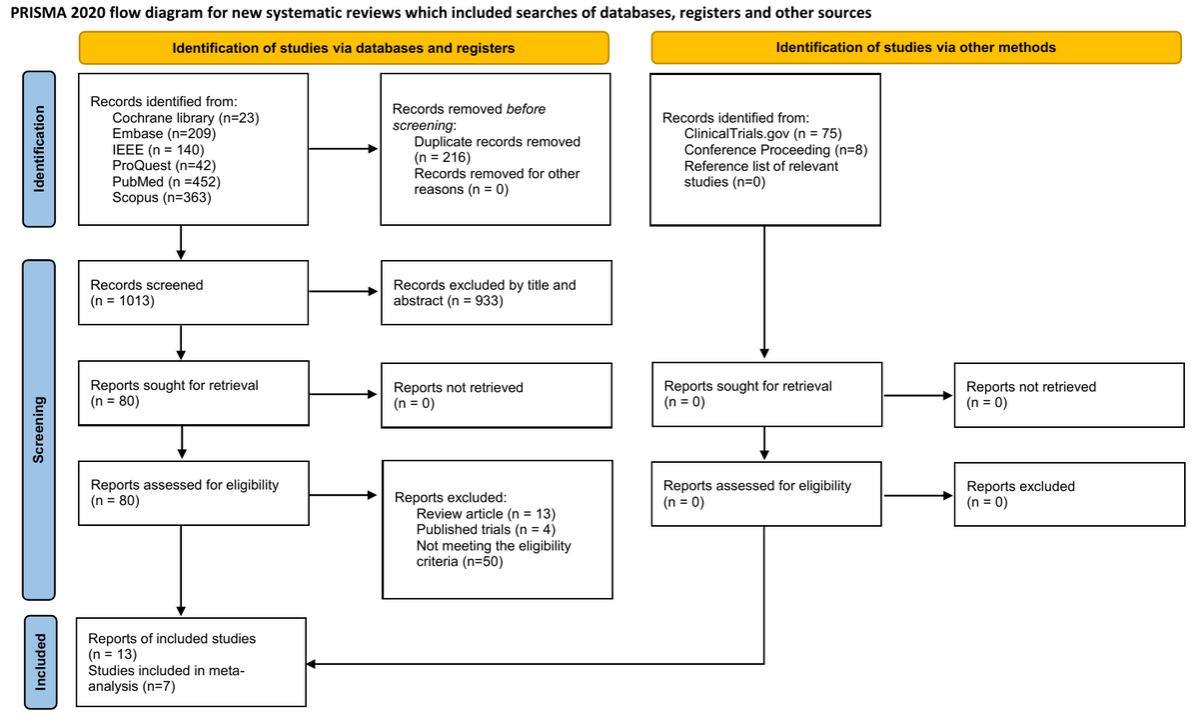
PRISMA flow diagram for the literature search. A total of 1229 records were retrieved from 8 databases and 1 record from citation searching. After removing 216 duplicates and screening titles or abstracts, 80 full texts were assessed. After 67 studies were excluded due to not meeting the criteria, 13 studies were included, with 7 studies providing sufficient data for meta-analysis. PRISMA: Preferred Reporting Items for Systematic Reviews and Meta-Analyses.

As illustrated in Figure 1, a total of 1229 records were retrieved from 8 electronic databases (Multimedia Appendix 2), with no additional records retrieved through other methods. After removing 216 duplicates, 1013 records remained for review. Title and abstract screening excluded 933 papers based on the predefined inclusion and exclusion criteria, resulting in 80 papers for full-text reviews. Of these, 67 were excluded because they did not meet the eligibility criteria (Multimedia Appendix 2). Ultimately, 13 RCTs were included in this review. The details of the search strategies are presented in Multimedia Appendix 2.

### Characteristics of Included Studies

The characteristics of the 13 included RCTs are shown in Table 1. All studies were published between 2013 and 2023 and were conducted in 6 countries: Canada, the United States, Italy, Iran, Turkey, and Taiwan. A total of 619 participants were enrolled (intervention group: 301 and control group: 318), with individual study sample sizes ranging from 11 to 103. Participants were aged 2-19 years, most of whom were of school age, and all were in pediatric hospital settings due to acute illness, chronic disease, or surgical procedures. Additionally, the settings in which the interventions were implemented were diverse. Two trials were conducted in emergency departments [42,43], 2 in surgical wards and operating rooms [44,45], 2 in oncology units or hematology clinics [46,47], 3 in pediatric wards [48-50], 1 in a postanesthesia care unit [51], 1 in a radiology department [52], 1 in a hospice unit [53], and 1 in a hospital-based game room [54].

**Table 1:** Characteristics of the included RCTs^a^, including author, publication year, country, study objectives, number of participants, participant characteristics, settings, measurements, and main results.

Author (year), country	Objectives	Number of participants (IG^b^/CG^c^)	Study population	Age (years)	Setting	Measurements	Main results
Alemi et al (2016) [46], Iran	Exploring the effect of SARs^d^ as a therapy-assistive tool	6/5	Children with cancer receiving active therapy	7-12	Oncology unit in the hospital	MASC^e^, CDI^f^, and CIA^g^	Improved anxiety, anger, and depression with emotional support.
Ali et al (2021) [43], Canada	Effect of SARs during the invasive procedure	43/43	Require intravenous insertion	6-11	Emergency department	FPS-R^h^ and OSBD-R^i^	Reduced distress; none in pain.
Beraldo et al (2019) [53], Italy	Potential of SARs during invasive medical procedures	14/14	Inpatients prepared for invasive procedures (eg, spinal tap)	3-19	Hospice unit in the hospital	Emotion questionnaire	Overall, reduced negative feelings, increased positive emotions. Most rated the experience positively.
Chang et al (2023) [48], Taiwan	Impact of SARs-assisted digital storytelling of intravenous procedure	26/26	Inpatients with intravenous access	5-10	Pediatric general ward in the hospital	MYPAS^j^	Reduced anxiety and improved therapeutic communication, emotions, and engagement.
Franconi et al (2023) [45], Italy	Potential of SARs during the preoperative preparation	30/30	Preparing to undergo surgery	2-14	Pediatric surgical ward and operating room in the hospital	CEMS^k^	The intervention group showed significantly lower anxiety levels.
Jibb et al (2018) [47], Canada	Impact of SARs during subcutaneous port access insertion	19/21	Children with cancer and a subcutaneous port underwent active therapy	4-9	Hematology clinic in a pediatric hospital	FPS-R, CFS^l^, and BAADS^m^	SARs were acceptable, but had no effect on pain or distress.
Lee-Krueger et al (2021) [44], Canada	Effect of SARs support during intravenous induction	45/58	Required intravenous insertion before surgery	4-12	Operating room in a pediatric hospital	FPS-R and CFS	No significant differences in pain or fear across groups.
Logan et al (2019) [49], United States	The feasibility and acceptability of SARs technology	13/16	Inpatient over 48 hours with cancer or surgery	3-10	General and hematology-oncology ward in a hospital	FPS-R, NRS^n^, FAS^o^,PANAS-C^p^, and STAI-C^q^	Children exposed to SARs reported more positive emotion. SARs were mostly acceptable.
Meghdari et al (2018) [54], Iran	Acceptability and involvement of SARs assistance	7/7	Children with cancer receiving active therapy	5-12	Game room in the hospital	TS-SF^r^ and SAM^s^	Revealed high engagement and interest of pediatric patients with cancer with the SARs.
Okita (2013) [50], United States	Potential of SARs companions and involvement with family	9/9	Hospitalized female children	6-16	General ward in a hospital	WBFPRS^t^ and STAI-C	Significant reduction in pain and anxiety when children and parents engaged with SARs together.
Rossi et al (2022) [42], Italy	Exploring the impact of SARs on stress before medical procedures	36/37	Waiting to access the medical office	3-10	Emergency department	Salivary cortisol levels and heart rate	Significant decrease in salivary cortisol levels and heart rate. The effect was stronger in girls.
Topçu et al (2023) [51], Turkey	Effect of SARs on the postoperative recovery	42/42	Underwent day surgery	5-10	Postanesthesia care unit in a hospital	CSA^u^	Significant group differences in postoperative anxiety and mobilization time.
Trost et al (2020) [52], United States	Impact of an empathic SARs during intravenous insertion	11/10	Required intravenous insertion before MRI^v^	4-14	Radiology department in a hospital	WBFPRS and CFS	Pain and fear significantly decreased over time.

^a^RCT: randomized controlled trial.

^b^IG: intervention group.

^c^CG: control group.

^d^SAR: socially assistive robot.

^e^MASC: Multidimensional Anxiety Children Scale.

^f^CDI: Children’s Depression Inventory.

^g^CIA: Children’s Inventory of Anger.

^h^FPS-R: Faces Pain Scale-Revised.

^i^OSBD-R: Observed Scale of Behavioral Distress-Revised.

^j^MYPAS: Modified Yale Preoperative Anxiety Scale.

^k^CEMS: Children’s Emotional Manifestation Scale.

^l^CFS: Child Fear Scale.

^m^BAADS: Behavioral Approach-Avoidance Scale.

^n^NRS: Numeric Rating Scale.

^o^FAS: Facial Affective Scale.

^p^PANAS-C: Positive and Negative Affect Scales for Children.

^q^STAI-C: State-Trait Anxiety Inventory for Children.

^r^TS-SF: Transportation Scale-Short Form.

^s^SAM: Self-Assessment Manikin Questionnaire.

^t^WBFPRS: Wong-Baker FACES Pain Rating Scale.

^u^CSA: children’s state anxiety.

^v^MRI: magnetic resonance imaging.

### Design of SARs Interventions and Comparators

The included interventions varied in terms of timing, frequency, and technological features (Table 2). Six studies implemented SARs before or during invasive procedures [43,44,47,48,52,53], 4 addressed broader hospital experience contexts [46,49,50,54], 2 focused on preoperative care [45] or postoperative care [51], and 1 was conducted before a noninvasive procedure [42]. The intervention duration ranged from 3 to 40 minutes; 11 studies used a single session, while 2 adopted repeated sessions [46,51]. SARs primarily provide distraction, cognitive behavioral strategies, and emotional companionship. Technical difficulties were reported in 4 studies [42,43,47,49], mainly due to connectivity or hardware malfunctions, with rates ranging from 9% (4/46) to 60% (26/43).

**Table 2:** Summary of interventions and comparators, including type of SARs^a^, characteristics of intervention design, type of comparators, duration of intervention, and technical difficulties.

Author (year)	Type of SARs	Interventions	Comparators	Duration	Follow-up	Technical difficulties
Alemi et al (2016) [46]	NAO	The hybrid-operated SARs engaged children through specific dialogue with a psychologist	Alternative intervention (only with a psychologist)	5 min	8 sessions	None reported
Ali et al (2021) [43]	NAO	The SARs were programmed with self-introduction, breathing guidance, and dance during intravenous insertion	Standard care	5-10 min	No	Occurred in 60% (26/43): connectivity, delays, tablet freezing, volume issues, shutdowns, or falls
Beraldo et al (2019) [53]	Pepper	The hybrid operative SARs interacted with dialogue, gestures, games, and music during invasive procedures	Alternative intervention (Sanbot robot)	Not reported	No	None reported
Chang et al (2023) [48]	Kebbi	Preprogrammed with digital storytelling during intravenous insertion	Standard care	40 min	No	None reported
Franconi et al (2023) [45]	NAO	Through hybrid operative programs of speech, singing, and play, and distracted attention before surgery	Standard care	Not reported	No	None reported
Jibb et al (2018) [47]	NAO	SARs were preprogrammed with CBT^b^ strategies such as deep breathing and encouragement during subcutaneous port insertion	Alternative intervention (active distraction with NAO)	7-10 min	No	35% (14/40): connection loss, phrase repetition
Lee-Krueger et al (2021) [44]	NAO	The SARs were preprogrammed to guide deep breathing exercises before intravenous induction for surgery	Standard care	5-20 min (mean 10 min)	No	None reported
Logan et al (2019) [49]	Huggable bear	Teleoperation to interact with children through speech, games, and touch	Alternative intervention (plush teddy bear)	9-40 min (mean 26 min)	No	9% (4/46): wireless interference, delays, malfunctions, and speaker failure
Meghdari et al (2018) [54]	Arash	Telling stories through preprogrammed dialogue, expression, and gesture	Alternative intervention (an audiobook with the same stories)	3 min	No	None reported
Okita (2013) [50]	Paro	Accompanied by mom and interacted with autonomous SARs through contact	Alternative intervention (alone with the SARs)	30 min	No	None reported
Rossi et al (2022) [42]	NAO	The hybrid SARs engaged children with songs, stories, jokes, and riddles before the medical procedure	Standard care	15 min	No	Background noise or mispronunciation required teleoperation
Topçu et al (2023) [51]	Macrobot	In postoperative recovery, autonomous SARs encouraged and accompanied children during mobilization	Alternative intervention (nurses)	4-10 min	3 sessions	None reported
Trost et al (2020) [52]	MAKI	During intravenous insertion, the SARs provided empathetic responses	Standard care	Not reported	No	None reported

^a^SAR: socially assistive robot.

^b^CBT: cognitive behavioral therapy.

Across the 13 included RCTs, 6 studies compared the SARs interventions with standard hospital care. The remaining 7 studies used diverse comparators, including psychologist-led therapy [46], another robotic platform [53], an alternative SARs-based distraction program [47], a plush teddy bear [49], audiobooks delivering the same narratives [54], being alone with the SARs [50], and nurse-led postoperative recovery [51]. These variations in comparator conditions illustrate the heterogeneity of approaches in contextualizing the role of SARs in pediatric care.

Nine types of SARs were used in the included studies (Table 3). Their physical appearances can be broadly categorized as humanoid (eg, NAO byAldebaran, Pepper bySoftBank, and Arash), animal-like (Huggable and Paro by National Institute of Advanced Industrial Science and Technology), or robot-like (Sanbot by Sanbot, Kebbi by Nuwa, MAKI, and Macrobot by Silverlit). Most SARs interacted with children using voice and gestures, and visual aids through camera input. Humanoid robots typically feature advanced functions, such as facial expression recognition and tactile feedback. The operational modes varied across autonomous, hybrid, and teleoperated systems. Cost information was available in only 2 studies: Arash (US $6000) [54] and MAKI (US $2985) [52]. The price of Macrobot (US $27-$78) [51] was obtained from commercial retail websites. For the other SARs, pricing information was obtained from the manufacturer’s specifications. Overall, 6 SARs were commercially available products, whereas Huggable and Arash were developed in research laboratories, and MAKI was custom-fabricated using 3D printing technology.

**Table 3:** Overview of SARs^a^, including cost, appearance, interaction features, technical specifications, and type of operation.

SARs	Cost (US $)	Appearance	Interaction features	Specifications	Type of operation
Arash [54]	6000	Humanoid (134 cm tall and 24 kg)	Voice, vision, facial expression, and gesture	Microphones, sensors, facial expression recognition, voice localization, camera, and screen	Preprogrammed automation
Huggable bear [49]	Not reported	Bear-like	Voice and gestures	Microphones, a camera, and fluffy	Teleoperated
Kebbi [48]	600	Robot-like (32 cm tall and 2.5 kg)	Voice, vision, and gesture	Microphones, camera, screen, and touch sensor	Preprogrammed automation
MAKI [52]	2985	Robot-like (34 cm tall and 2 kg)	Voice	Microphones, speech recognition, text-to-speech, and lights	Teleoperated
Macrobot [51]	27-78	Robot-like (20 cm tall and 0.25 kg)	Gestures and people following	Obstacle sensor, battery-powered, and wheel	Automation
NAO [42-47]	7500-13,000	Humanoid (57 cm tall and 5.5 kg)	Voice, vision, and gestures	Microphones, camera, LED, text-to-speech, and face detection	Hybrid
Paro [50]	6000	Seal-like (57 cm length and 2.7 kg)	Body movements react to stroking and cuddling	Microphones, fluffy, and touch sensor	Automation
Pepper [53]	32,000-49,900	Humanoid (120 cm tall and 28 kg)	Voice, vision, gestures, animations, and people detection	Microphones, cameras, LED, touch sensors, and tablet screen	Hybrid
Sanbot [53]	8500	Robot-like (90 cm tall and 19 kg)	Voice, vision, gestures, people detection and following, and animations	Microphones, cameras, LED, touch sensors, screen, and laser projector	Hybrid

^a^SAR: socially assistive robot.

### Risk of Bias and GRADE Assessment

**Figure 2:**
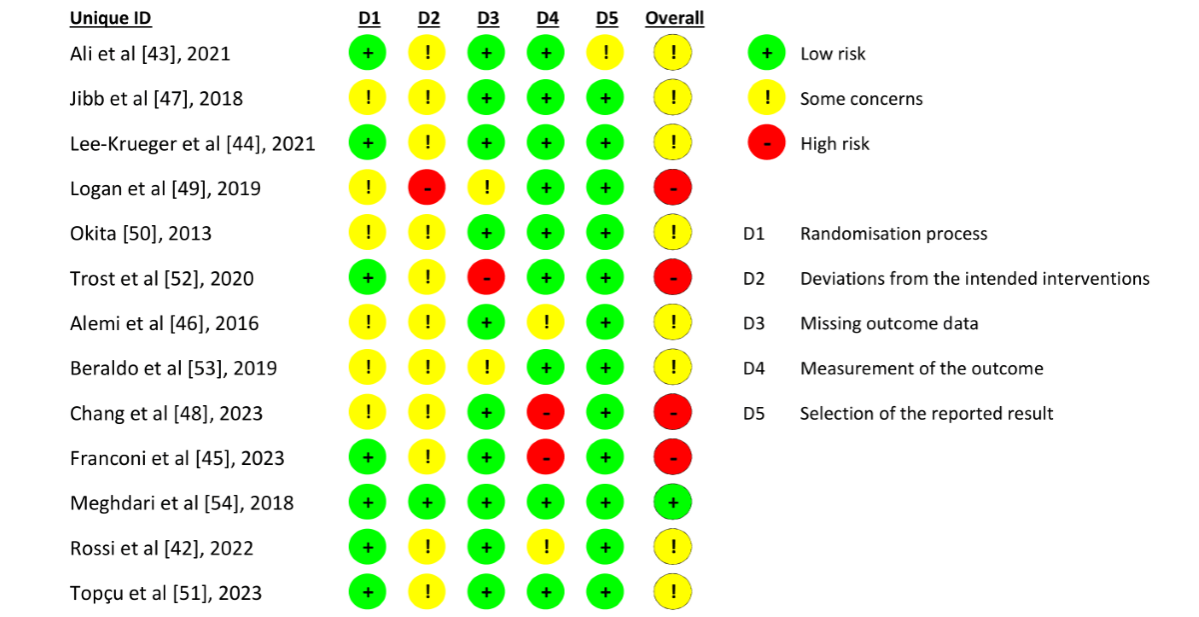
Summary of risk of bias assessments across 13 included RCTs [42-54]. The risk of bias was evaluated across 5 domains. Most of the studies were identified as having some concerns, with deviations from the intended interventions (domain 2) being the most prevalent source of bias. D: domain; RCT: randomized controlled trial.

Eight studies were assessed as having some concerns regarding the overall risk of bias [42-44,46,47,50,51,53], and 4 were assessed as having a high risk of bias [45,48,49,52]. The most frequent high-risk domains were deviations from the intended interventions (domain 2) and measurement of the outcome (domain 4; Figure 2). As the SARs intervention could not be blinded, some concerns were particularly identified in domain 2, where 1 trial [49] was rated as high risk because its control group may have had an active role beyond that of passive control, potentially influencing the comparison with the intervention group. Two other studies were rated as high risk in domain 4 because the individuals assessing the outcomes also participated in the intervention, which may have introduced observer bias [45,48]. Additionally, 1 trial was rated as having a high risk of missing outcome data because it did not report 2 missing participants [52].

According to the GRADE assessment, all outcomes were rated as moderate-certainty evidence (Multimedia Appendix 2). Pain reduction showed moderate-certainty evidence when compared with both standard and alternative care. Anxiety and fear reduction were also rated as moderate, indicating potential benefits but inconclusive effects. Distress reduction was similarly rated as moderate, supported by a single trial. Overall, these outcomes are considered clinically important; however, the certainty of evidence was limited by the risk of bias and the small number of studies.

The risk of bias was evaluated across 5 domains. Most of the studies were identified as having some concerns, with deviations from the intended interventions (domain 2) being the most prevalent source of bias.

### Narrative Synthesis

The outcomes of the 13 studies varied by domain (Table 4). For primary pain level measures in 6 studies, significant reductions were observed in 1 study [50], whereas the other 5 [43,44,47,49,52] reported no significant differences, reflecting mixed evidence regarding the analgesic benefits of SARs. As participant and personnel blinding were unfeasible in SARs interventions, 4 trials were rated with some concerns, and 2 were high-risk in reporting bias and comparator response bias. Secondary emotion-related outcomes were anxiety, fear, distress, emotional engagement, state positive and negative emotion, and stress level. Stress-related physiological outcomes were more consistent across 1 trial, which demonstrated significant decreases in both salivary cortisol and heart rate [42]. Anxiety outcomes showed clearer benefits, with 6 studies reporting significant reductions [45,46,48,50,51,53], while studies had some concerns or a high risk of bias due to observer bias. Three studies reported null effects of fear [44,52,53]. Of the 2 studies [43,47], only 1 reported a significant reduction in distress [43]. For state emotions, SARs enhanced emotional engagement and positive emotions in 2 studies [49,53]. Additionally, 2 studies documented greater engagement with SARs and narrative immersion [48,54]. Detailed statistical findings of each study are presented in Multimedia Appendix 2.

**Table 4:** Summary of statistical results across studies, including pain, anxiety, fear, distress, stress, and emotional engagement outcomes.

Author (year)	Pain	Anxiety	Fear	Distress	Stress	Emotional engagement
Alemi et al (2016) [46]	NA^a^	↓^b^ (*P*=.002)	NA	NA	NA	NA
Ali et al (2021) [43]	NS^c^ (*P*=.13)	NA	NA	↓ (*P*=.047)	NA	NA
Beraldo et al (2019) [53]	NA	↓ (*P*=.047)	NS (*P*=.06)	NA	NA	NA
Chang et al (2023) [48]	NA	↓ (*P*<.05)	NA	NA	NA	↑^d^ (*P*<.05)
Franconi et al (2023) [45]	NA	↓ (*P*=.03)	NA	NA	NA	NA
Jibb et al (2018) [47]	NS (*P*=.07)	NA	NA	NS (*P*=.06)	NA	NA
Lee-Krueger et al (2021) [44]	NS (*P*=.98)	NA	NS (*P*=.33)	NA	NA	NA
Logan et al (2019) [49]	NS^e^	NA	NA	NA	NA	NA
Meghdari et al (2018) [54]	NA	NA	NA	NA	NA	↑ (*P*<.03)
Okita (2013) [50]	↓ (*P*<.001)	↓ (*P*<.01)	NA	NA	NA	NA
Rossi et al (2022) [42]	NA	NA	NA	NA	↓ (*P*<.01)	NA
Topçu et al (2023) [51]	NA	↓ (*P*=.005)	NA	NA	NA	NA
Trost et al (2020) [52]	NS (*P*=.758)	NA	NS (*P*=.472)	NA	NA	NA

^a^NA: outcome not assessed.

^b^↓: significant decrease.

^c^NS: nonsignificant.

^d^↑: significant increase.

^e^The exact *P* value was not reported in the original study.

### Meta-Analysis

Among the 13 included studies, 7 met the criteria for this meta-analysis, involving a total of 359 participants. Pain was the primary outcome, whereas anxiety, fear, and distress were secondary emotional responses (Table 5). All pooled estimates were calculated using the Hartung-Knapp-Sidik-Jonkman random-effects method, and PIs were displayed on the forest plots, except for outcomes with very few included studies, such as fear and distress. Funnel plots were generated for pain and anxiety to provide a visual assessment for small-study effect (Multimedia Appendix 2). As the number of included studies was very limited (pain, n=5; anxiety, n=3; distress, n=2; and fear, n=2), no Egger tests were conducted [40].

**Table 5:** Summary of data extraction as mean (SD) from 7 studies in the meta-analysis, including outcomes: pain, anxiety, fear, and distress.

Author (year)	Pain		Anxiety		Fear		Distress	
	IG^a^	CG^b^	IG	CG	IG	CG	IG	CG
Alemi et al (2016) [46], mean (SD)	NA^c^	NA	1.89 (0.20)	2.38 (0.43)	NA	NA	NA	NA
Ali et al (2021) [43], mean (SD)	2.71 (2.96)	3.74 (3.08)	NA	NA	NA	NA	0.78 (1.32)	1.49 (2.36)
Jibb et al (2018) [47], mean (SD)	1.00 (2.30)	1.40 (3.00)	NA	NA	NA	NA	1.60 (1.30)	1.40 (0.80)
Lee-Krueger et al (2021) [44], mean (SD)	2.74 (2.96)	2.76 (2.97)	NA	NA	1.13 (1.02)	1.16 (1.26)	NA	NA
Okita (2013) [50], mean (SD)	2.78 (1.92)	5.13 (2.30)	1.64 (0.31)	2.81 (0.53)	NA	NA	NA	NA
Topçu et al (2023) [51], mean (SD)	NA	NA	2.74 (2.6)	4.5 (2.96)	NA	NA	NA	NA
Trost et al (2020) [52], mean (SD)	1.55 (0.30)	2.47 (0.40)	NA	NA	1.80 (1.33)	2.10 (0.76)	NA	NA

^a^IG: intervention group.

^b^CG: control group.

^c^NA: outcome not assessed.

### Pain

**Figure 3:**
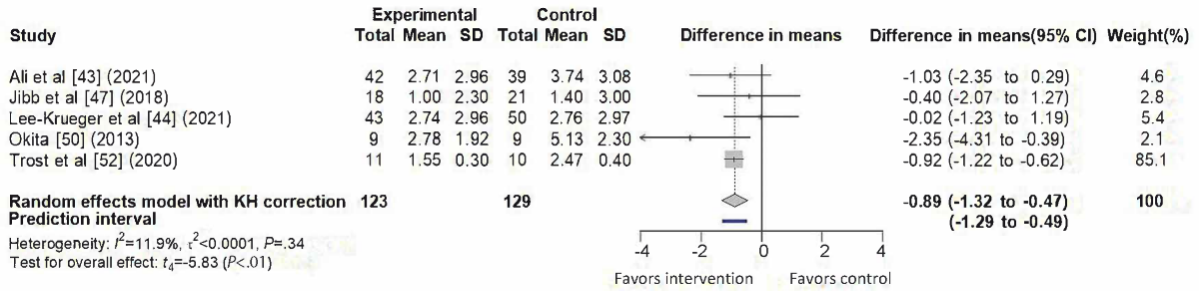
Forest plot of the effect on pain outcomes [43,44,47,50,52]. KH: Knapp-Hartung correction.

A total of 5 studies [43,44,47,50,52] contributed data to the meta-analysis of pain outcomes, as illustrated in Figure 3. The pooled analysis demonstrated a significant reduction favoring SARs interventions (difference in means=–0.89, 95% CI –1.32 to –0.47; 95% PI –1.29 to –0.49), with low heterogeneity (*I*²=11.9%, τ² < 0.0001, τ<0.01, *P*=.34). One study [52] contributed the largest weight (85.1%), attributable to its smaller variance. The funnel plot showed slight asymmetry (Multimedia Appendix 2).

### Anxiety

**Figure 4:**
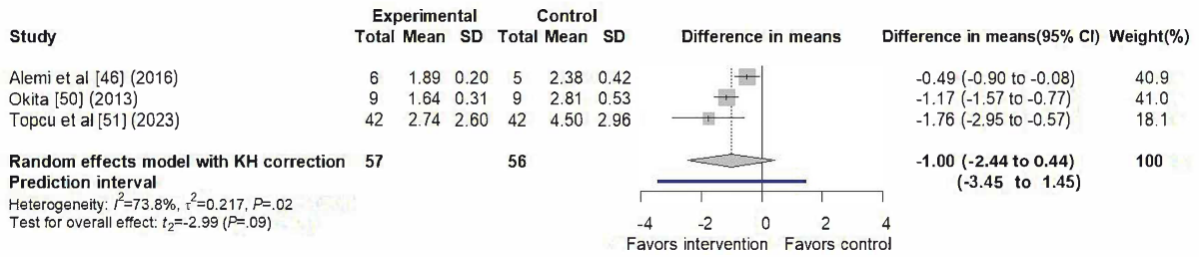
Forest plot of the effect on anxiety [46,50,51]. KH: Knapp-Hartung correction.

A total of 3 studies [46,50,51] contributed to the meta-analysis of anxiety outcomes, as illustrated in Figure 4. The random-effects model yielded a nonsignificant pooled effect (difference in means=–1.00, 95% CI –2.44 to 0.44; 95% PI –3.45 to 1.45), with substantial heterogeneity (*I*²=73.8%, τ²=0.2172, τ=0.466, *P*=.02). The funnel plot appeared symmetrical (Multimedia Appendix 2).

### Fear

**Figure 5:**
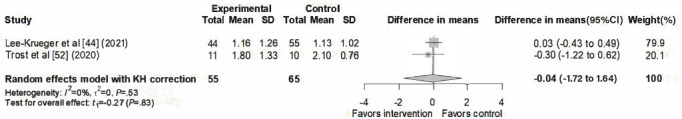
Forest plot of the effect on fear [44,52]. KH: Knapp-Hartung correction.

A total of 2 studies [44,52] contributed to the meta-analysis of fear outcomes, as illustrated in the forest plot (Figure 5). The pooled analysis showed no significant effect of SARs interventions (difference in means=–0.04, 95% CI –1.72 to 1.64), with no detected heterogeneity (*I*²=0%, τ²=0, *P*=.53).

### Distress

**Figure 6:**
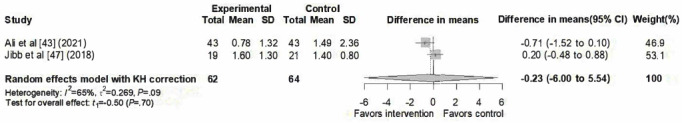
Forest plot of the effect of distress [43,47]. KH: Knapp-Hartung correction.

A total of 2 studies [43,47] were in the meta-analysis of distress outcomes, as illustrated in Figure 6. The pooled analysis showed no significant effect of SARs interventions (difference in means=–0.23, 95% CI –6.00 to 5.54) with substantial heterogeneity (*I*²=65%, τ²=0.2693, τ=0.519, *P*=.09).

In summary, the meta-analysis provides evidence that SARs interventions may effectively reduce pain for children in the hospital. By contrast, the findings for anxiety, fear, and distress remain inconclusive due to nonsignificant pooled effects and considerable heterogeneity across studies.

## Discussion

### Principal Findings

This systematic review and meta-analysis synthesized evidence from 13 RCTs to evaluate the effectiveness of SARs in reducing pain and emotional outcomes, including anxiety, fear, and distress, among pediatric patients in hospital settings. Beyond the meta-analysis, our review conducted a comprehensive narrative analysis, integrating intervention characteristics and contextual factors to provide an understanding of real-world clinical implementation and future research design. Overall, the pooled analysis suggested that SARs interventions may offer beneficial effects for pain reduction, whereas their impact on emotional outcomes was not statistically significant. However, these findings should be interpreted with caution, given the presence of some concerns and high risks of bias in several domains, as well as the overall moderate certainty of evidence. Importantly, these results have practical relevance for health care providers and researchers, offering insights for future clinical implementation and study design aimed at adopting SARs as child-friendly and effective adjuncts in pediatric hospital care.

### Pain

SARs interventions demonstrated a statistically significant reduction in children’s pain, providing moderate-certainty evidence that such interventions may help alleviate pain in hospital settings. Among the 5 studies synthesized, 1 trial [52] was rated as high risk due to reporting bias and lack of blinding, while the others were rated as having some concerns. Notably, this high-risk study accounted for a large weight in the meta-analysis, suggesting that the pooled effect for pain may be disproportionately influenced by it and should therefore be interpreted with caution.

The PI was slightly narrower than, but consistent with, the effect of the CI. As prior studies [39,55], a narrower PI may indicate low between-study heterogeneity, which in this study could also reflect the large weighting of a single trial influencing the pooled estimate and reducing observed variability. This pattern suggests that similar beneficial effects may be observed under comparable conditions, but the limited evidence base warrants a conservative interpretation of these findings.

From a clinical perspective, these results imply that when intervention protocols, implementation settings, and participant characteristics are similar, clinicians may expect consistent and meaningful pain reduction with the use of SARs. In practice, SARs can provide distraction, emotional support, and engagement as adjuncts to standard pain management strategies. The combination of a statistically robust pooled effect and PI offers moderate yet credible evidence that SARs can reduce children’s pain perceptions during hospital-based procedures.

However, the duration of SARs interventions varied considerably across studies, revealing a lack of standardization in exposure time. Due to this variability, a dose-response relationship between intervention length and pain reduction could not be established. While short, single-session interventions may be well-suited for acute procedural pain, current evidence remains insufficient to confirm sustained benefits for children undergoing longer hospital stays. Collectively, these findings position SARs as promising, child-friendly adjuncts within multimodal pediatric pain management, though further methodologically rigorous and well-powered RCTs are needed to consolidate their clinical credibility, optimize implementation protocols, and determine long-term therapeutic potential.

### Anxiety, Fear, and Distress

The emotional outcomes revealed a more complex and context-dependent pattern compared with the primary pain outcomes. Among the studies included in this review, SARs interventions appeared effective in reducing children’s anxiety when both self-reported and observer-rated measures were considered. However, the meta-analysis, which primarily focused on children’s self-reported anxiety scales, did not yield a statistically significant pooled effect. This divergence is likely attributable to differences in outcome measurement. Previous meta-analyses [22-24] reported significant reductions in anxiety, which typically combined observer-rated assessments with children’s self-reports, whereas our analysis distinguished between the two. This distinction reflects that anxiety, as an inherently subjective emotional experience, is best captured through the individual’s own perspective [25,26]. The nonsignificant result observed in our analysis aligns with prior evidence showing discrepancies between observer- and self-reported measures [56], underscoring the need for further investigation into how these differing perspectives capture children’s emotional experiences. The overall moderate certainty of evidence reflects methodological limitations identified in the included trials, particularly the risk of bias from the nonblinded nature, inadequate statistical power, and reporting bias.

Furthermore, the CI reflects the average effect in this meta-analysis, while the wide PI illustrates the likely variation in true effects in future studies and clinical contexts [39,55]. The wide PI observed for anxiety suggests that the true effects of SARs may vary substantially across clinical contexts, indicating that while some settings may observe meaningful emotional benefits, others may experience null or even opposite effects. The statistical heterogeneity for anxiety and distress can be attributed to significant methodological and clinical context differences across the included trials. The studies varied widely in their clinical settings, study populations, intervention designs, and the specific features of SARs. Such variability likely reflects differences between included studies, rather than inconsistency in the underlying potential of SARs. This highlights the importance of contextual and implementation factors in shaping the emotional outcomes of SARs interventions. However, due to the limited number of studies, these findings should be interpreted with caution.

These contextual variations suggest that the effectiveness of SARs may be highly specific to a particular population, clinical context, or interaction mode. From a practical perspective, these findings emphasize the need for an approach grounded in real-world clinical contexts to ensure effective and meaningful integration of SARs into patient care. Overall, the evidence of SARs deployment for emotional support in pediatric hospital settings was limited, highlighting the need for more standardized trials to address these methodological and contextual variations.

### Clinical and Practical Implications

The evidence from this review indicates that SARs represent an engaging and child-friendly adjunct for pain management in pediatric hospital settings. Our pooled results demonstrated a statistically significant reduction in pain, and the PI suggested that these benefits may be reproducible in similar clinical contexts. However, the current evidence for emotional outcomes remains limited and heterogeneous, emphasizing the need for caution in their implementation for psychosocial support.

The successful integration of SARs into clinical practice necessitates careful consideration of feasibility, ethical implications, and long-term sustainability. Clinically, SARs function primarily as assistants, supporting but not replacing human caregivers. Therefore, effective implementation requires comprehensive staff training in interaction protocols and hygiene management, alongside strong institutional support to ensure appropriate use and maximize clinical benefits. In addition, reliable technical support and regular maintenance are essential to sustain functionality, particularly in hospital settings that may have limited access to specialized technological personnel.

From an institutional perspective, performing a thorough cost-effectiveness analysis is essential. The initial acquisition costs of the SARs varied greatly and needed to be considered alongside the ongoing maintenance costs of hardware and software. A strategic evaluation of cost-effectiveness involving the adoption of innovative technologies, beginning with pilot studies to assess clinical feasibility before expanding to broader use, can further facilitate the full integration of SARs into health care settings.

### Ethical Considerations

Ethical dimensions are critical for the implementation of SARs in pediatric hospital care, particularly regarding safety, privacy, and autonomy [27,29]. Only 4 of the 13 included studies addressed ethical considerations, primarily focusing on children’s physical and psychological safety [46,49,53,54]. The evidence currently offers limited insight into the broader ethical dimensions of human-robot interaction. Therefore, we expanded upon these critical ethical considerations.

Beyond safety, privacy is a crucial issue, requiring secure data storage, parental consent, and adherence to data protection standards [28,57,58]. Psychological considerations and autonomy also warrant attention, while a few children may experience fear or negative experiences [12,27]. While SARs can provide comfort and support, some children may experience fear or discomfort [13,27,59]. These risks intersect with the question of autonomy, particularly as children’s interactions with robots may influence their social and emotional development.

The automation level of SARs varied across included studies; notably, 11 trials used hybrid or operator-guided systems. Such approaches may represent the safest balance between technological novelty and patient safety in current clinical practice [17,27,59,60].

### Strengths and Limitations

The primary strength of this review lies in its rigorous, systematic approach, coupled with the innovative integration of comprehensive contextual synthesis, cost-effectiveness, and ethical dimensions. The meta-analysis also allowed us to quantify and interpret the effect of SARs statistically. These contribute a framework for understanding SARs’ application relevant to real clinical practice.

However, several limitations should be acknowledged. The heterogeneity in methodological designs across included studies constrained the comparability of findings. The limited number of eligible trials presents a significant methodological constraint to performing subgroup analyses, particularly concerning statistical power. Although funnel plots were conducted to visually assess potential asymmetry, the small number of eligible trials constrained the reliable assessment of small-study effects (Egger test), as statistical power is limited with few studies [40]. Last, the moderate certainty of evidence underscores the need for greater methodological rigor in future research. In summary, these factors suggest that while the findings offer meaningful insights, they should be interpreted with appropriate caution and contextual awareness.

### Future Research Directions

To address the risk of bias concerns identified in this review, future RCTs should adhere to rigorous methodological and reporting standards. Larger, well-designed, and adequately powered studies are warranted to reduce imprecision and enhance generalizability. As participant and personnel blinding are inherently unfeasible in SARs interventions, alternative strategies are suggested to minimize observer and response bias. These may include the use of blinded outcome assessors, standardized intervention protocols, and integrating objective indicators (eg, physiological parameters, objective behavioral indicators, speech emotion recognition, or facial expression recognition) to mitigate human influence during assessment.

As pain and emotions are inherently subjective experiences, self-reported measures remain the most direct indicators. However, combining validated self-report instruments with objective or observer-based assessments may provide a more comprehensive and balanced understanding. Transparent reporting of contextual and procedural factors will further facilitate comparability and reproducibility.

Moreover, research may expand beyond mitigating negative emotions to explore how SARs promote positive emotional responses and evaluate multisession interventions to determine sustained effects. Technological development is also crucial for improving system robustness, minimizing technical failures, and enhancing the usability of the operation. Notably, integrating ethical considerations, including child autonomy, privacy, and data protection, is essential for responsible future research.

### Conclusion

This systematic review and meta-analysis suggest that SARs have potential as a valuable adjunct for pain management in pediatric hospital care. The observed reduction in pain across comparable clinical contexts indicates that SARs can provide consistent and clinically meaningful benefits when appropriately implemented. In contrast, the evidence for their effects on emotional outcomes remains ambiguous. The wide PI observed for anxiety suggests that the effects of SARs may vary substantially across clinical contexts, while some children may experience emotional benefits, others may show null or even opposite effects, highlighting the important role of contextual factors of SARs implementation. The overall concerns of risk of bias underscore the need for methodological rigor in future research to consolidate the evidence base.

At present, SARs can be regarded as a promising nonpharmacological tool for pain management. Their ethical and effective integration into pediatric practice requires adherence to clear principles that prioritize child-friendly care. Moving forward, research should combine technological innovation with psychosocial intervention design to evaluate the cumulative effects of multisession SARs interactions and to explore their potential to enhance positive emotions, engagement, and resilience. Through such evidence-driven and ethically grounded development, SARs may evolve into a vital component of child-centered digital health, fostering more positive and supportive health care experiences for children.

## Data Availability

All data analyzed in this study are included in the paper. Further details are available from the corresponding author upon reasonable request.

## Appendix

Multimedia Appendix 1 PRISMA checklist.

Multimedia Appendix 2 Additional information regarding study search, inclusion, and exclusion, statistics, funnel plots, and a summary of the certainty of evidence assessment.

## Abbreviations

GRADE: Grading of Recommendations, Assessment, Development, and Evaluation
PI: prediction interval
PICO: Population, Intervention, Comparison, and Outcomes
PRESS: Peer Review of Electronic Search Strategies
PRISMA: Preferred Reporting Items for Systematic Reviews and Meta-Analyses
PRISMA-S: Preferred Reporting Items for Systematic Reviews and Meta-Analyses Literature Search Extension
PROSPERO: International Prospective Register of Systematic Reviews
RCT: randomized controlled trial
SAR: socially assistive robot
