# Supplementary material for: Socially Assistive Robots for Pain Management and Emotional Responses in Pediatric Hospital Care: Systematic Review and Meta-Analysis
Source: J Med Internet Res. 2025 Nov 26;27:e76427. doi: 10.2196/76427 (PMC12696453; doi:10.2196/76427)

**Table S1.** Summary of five platforms and eight electronic databases.

| Platforms | Electronic databases |
| --- | --- |
| Elsevier | Embase, Scopus |
| IEEE Xplore | IEEE Xplore Digital Library |
| NLM | PubMed |
| ProQuest | Health & Medical Collection, MEDLINE, ProQuest Dissertations & Theses A&I |
| Wiley | Cochrane Library |

**Table S2.** Search strategies and the date of each platform (Initial search on May 6, 2025, and updated on October 7, 2025).

| Platform (search date) | Database | No | Query | Results |
| --- | --- | --- | --- | --- |
| IEEE Xplore (2025/10/7) | IEEE Xplore digital library | 1 | ("All Metadata":child*) AND ("All Metadata":hospital*) | 4,974 |
|  |  | 2 | ("All Metadata":pediatric) OR ("All Metadata":pediatrics) AND ("All Metadata":inpatient*) | 3,468 |
|  |  | 3 | ("All Metadata":robot*) | 386,233 |
|  |  | 4 | ("All Metadata":pain) OR ("All Metadata":painful) OR ("All Metadata":distress*) OR ("All Metadata":fear) OR ("All Metadata":fearful) OR ("All Metadata":anxi*) OR ("All Metadata":emot*) OR ("All Metadata":well-being) OR ("All Metadata":well being) | 796,880 |
|  |  | 5 | ("All Metadata":autis*) OR ("All Metadata":cerebral palsy) | 5,152 |
|  |  | 6 | ((((((All Metadata:child*) AND (All Metadata:hospital*))) OR ((All Metadata:pediatric) OR (All Metadata:pediatrics) AND (All Metadata:inpatient*))) AND ((All Metadata:robot*))) AND ((All Metadata:pain) OR (All Metadata:painful) OR (All Metadata:distress*) OR (All Metadata:fear) OR (All Metadata:fearful) OR (All Metadata:anxi*) OR (All Metadata:emot*) OR (All Metadata:well-being) OR (All Metadata:well being))) NOT ((All Metadata:autis*) OR (All Metadata:cerebral palsy)) | **141** |
| National Library of Medicine (2025/10/7) | PubMed | #1 | "child, hospitalized"[MeSH Terms] OR ("hospital*"[All Fields] AND "child*"[All Fields]) | 1,373,524 |
|  |  | #2 | ("inpatients"[MeSH Terms] OR "inpatient*"[All Fields]) AND ("pediatric*"[All Fields] OR "paediatric*"[All Fields] OR "pediatrics"[MeSH Terms]) | 17,247 |
|  |  | #3 | "robotics"[MeSH Terms] OR "robotics"[All Fields] OR ("social"[All Fields] AND "robot"[All Fields]) OR "social robot"[All Fields] OR "robotics"[MeSH Terms] | 78,879 |
|  |  | #4 | "pain"[MeSH Terms] OR "emotions"[MeSH Terms] OR "fear"[MeSH Terms] OR "anxiety"[MeSH Terms] OR "psychological well being"[MeSH Terms] OR "distress*"[All Fields] OR "emot*"[All Fields] OR "anxiet*"[All Fields] OR "health"[MeSH Terms] OR "health"[All Fields] OR "well"[All Fields] OR "well being"[All Fields] OR "wellbeing"[All Fields] | 11,546,162 |
|  |  | #5 | "autistic disorder"[MeSH Terms] OR "autistic disorder"[MeSH Terms] OR "cerebral palsy"[MeSH Terms] | 54,145 |
|  |  | #6 | ((("child, hospitalized"[MeSH Terms] OR ("hospital*"[All Fields] AND "child*"[All Fields])) OR (("inpatients"[MeSH Terms] OR "inpatient*"[All Fields]) AND ("pediatric"[All Fields] OR "paediatric"[All Fields] OR "pediatrics"[MeSH Terms]))) AND (("robotics"[MeSH Terms]) OR ("robot*"[All Fields])) AND ("pain"[MeSH Terms] OR "emotions"[MeSH Terms] OR "fear"[MeSH Terms] OR "anxiety"[MeSH Terms] OR "psychological well being"[MeSH Terms] OR "distress*"[All Fields] OR "emot*"[All Fields] OR "anxiet*"[All Fields] OR "well*"[All Fields])) NOT ("autistic disorder"[MeSH Terms] OR "ASD"[All Fields] OR "cerebral palsy"[MeSH Terms] OR "Aged"[All Fields] OR "adult"[All Fields]) | **452** |
| Elsevier (2025/10/7) | Embase^a^ | #1 | ('child'/exp OR 'hospitalized child'/exp) AND ('social robot'/exp OR 'robotics'/exp) AND ('pain'/exp OR 'emotion'/exp OR 'anxiety'/exp OR 'fear'/exp OR 'wellbeing'/exp OR 'distress syndrome'/exp OR 'distress') | **209** |
| Wiley (2025/10/7) | Cochrane library | #1 | ("Child"):ti,ab,kw AND (hospital*) | 48,192 |
|  |  | #2 | (inpatient):ti,ab,kw AND (pediatric):ti,ab,kw | 885 |
|  |  | #3 | ("robotics"):ti,ab,kw | 2,258 |
|  |  | #4 | (pain):ti,ab,kw OR (distress):ti,ab,kw OR (fear):ti,ab,kw OR (anxiety):ti,ab,kw OR (emotion):ti,ab,kw | 404,055 |
|  |  | #5 | ("well being"):ti,ab,kw | 24,615 |
|  |  | #6 | ((inpatient):ti,ab,kw AND (pediatric):ti,ab,kw OR ("Child"):ti,ab,kw AND (hospital*)) AND ("robotics"):ti,ab,kw AND ((pain):ti,ab,kw OR (distress):ti,ab,kw OR (fear):ti,ab,kw OR (anxiety):ti,ab,kw OR (emotion):ti,ab,kw OR ("well being"):ti,ab,kw) | **23** |
| Elsevier (2025/10/7) | Scopus | 1 | ( TITLE-ABS-KEY ( child* ) AND TITLE-ABS-KEY ( hospital* ) ) | 477,101 |
|  |  | 2 | ( TITLE-ABS-KEY ( inpatient* ) AND TITLE-ABS-KEY ( pediatric* ) OR TITLE-ABS-KEY ( paediatric* ) ) | 13,067 |
|  |  | 3 | TITLE-ABS-KEY ( robot* ) | 783,935 |
|  |  | 4 | ( TITLE-ABS-KEY ( pain* ) OR TITLE-ABS-KEY ( distress* ) OR TITLE-ABS-KEY ( emot* ) OR TITLE-ABS-KEY ( fear* ) OR TITLE-ABS-KEY ( anxi* ) OR TITLE-ABS-KEY ( wellbeing ) OR TITLE-ABS-KEY ( "well being" ) ) | 3,913,565 |
|  |  | 5 | ( TITLE-ABS-KEY ( autis* ) OR TITLE-ABS-KEY ( cerebral palsy ) ) | 198,243 |
|  |  | 6 | ( ( TITLE-ABS-KEY ( child* ) AND TITLE-ABS-KEY ( hospital* ) ) ) OR ( ( TITLE-ABS-KEY ( inpatient* ) AND TITLE-ABS-KEY ( pediatric* ) OR TITLE-ABS-KEY ( paediatric* ) ) ) AND ( TITLE-ABS-KEY ( robot* ) ) AND ( ( TITLE-ABS-KEY ( pain* ) OR TITLE-ABS-KEY ( distress* ) OR TITLE-ABS-KEY ( emot* ) OR TITLE-ABS-KEY ( fear* ) OR TITLE-ABS-KEY ( anxi* ) OR TITLE-ABS-KEY ( wellbeing ) OR TITLE-ABS-KEY ( "well being" ) ) ) AND NOT ( ( TITLE-ABS-KEY ( autis* ) OR TITLE-ABS-KEY ( cerebral palsy ) ) ) | **363** |
| ProQuest (2025/10/7) | Health & Medical Collection, MEDLINE, ProQuest Dissertations & Theses A &I | S1 | anywhere(child) AND anywhere(hospital*) | 2,555,425 |
|  |  | S2 | mesh(inpatients) AND mesh(pediatrics) | 168 |
|  |  | S3 | anywhere(robotics) | 225,815 |
|  |  | S4 | mesh(pain) OR mesh(emotion) OR mesh(fear) OR mesh(anxiety) OR mesh(psychological well being) OR subject(distress) | 866,311 |
|  |  | S5 | S1 OR S2  anywhere(child) AND anywhere(hospital*) OR mesh(inpatients) AND mesh(pediatrics) | 2,555,478 |
|  |  | S6 | S3 AND S5  anywhere(robotics) AND (anywhere(child) AND anywhere(hospital*) OR mesh(inpatients) AND mesh(pediatrics)) | 18,989 |
|  |  | S7 | S4 AND S6  (mesh(pain) OR mesh(emotion) OR mesh(fear) OR mesh(anxiety) OR mesh(psychological well being) OR subject(distress)) AND (anywhere(robotics) AND (anywhere(child) AND anywhere(hospital*) OR mesh(inpatients) AND mesh(pediatrics))) | **42** |
| National Library of Medicine (2025/10/7) | ClinicalTrials.gov | 1 | [Filter] Condition: child, [Filter] Intervention: robot, [Filter] Age: 0-19 | **75** |

^a^ The PICO search framework was used with terms entered in each PICO element

**Table S3.** List of excluded full-text reports and reasons for exclusion.

| Author (year) | Title | Review article | Published trials | Not meeting the eligibility criteria |
| --- | --- | --- | --- | --- |
| Alemi et al., (2014) | Effect of utilizing a humanoid robot as a therapy-assistant in reducing anger, anxiety, and depression |  |  | * |
| Ali et al., (2019) | Humanoid robot-based distraction to reduce pain and distress during venipuncture in the pediatric emergency department: A randomized controlled trial |  | * |  |
| Alves et al., (2024) | Supportive Technologies and Videogames for Pediatric Hospital Patients: A scoping review | * |  |  |
| Azmi et al., (2022) | Interactive Remote Robot for Pediatric Patients |  |  | * |
| Barco et al., (2013) | A robotic therapy for children with TBI |  |  | * |
| Baumann et al., (2023) | People Do Not Always Know Best: Preschoolers’ Trust in Social Robots |  |  | * |
| Beran et al., (2020) | Perspectives of child life specialists after many years of working with a humanoid robot in a pediatric hospital: Narrative design |  |  | * |
| Beran et al., (2021) | Implementation of a Humanoid Robot as an Innovative Approach to Child Life Interventions in a Children’s Hospital: Lofty Goal or Tangible Reality? |  |  | * |
| Beran et al., (2013) | Reducing children's pain and distress towards flu vaccinations: A novel and effective application of humanoid robotics |  |  | * |
| Betty, 2023) | Promoting Executive Function Through Computational Thinking and Robot: Two Studies for Preschool Children and Hospitalized Children |  |  | * |
| Beyer-Wunsch & Reichstein, 2020) | Effects of a humanoid robot on the well-being for hospitalized children in the pediatric clinic - An experimental study |  |  | * |
| Brenda Kimbembi Maleco et al., (2021) | Reducing negative emotions in children using social robots: systematic review | * |  |  |
| Castiglioni et al., (2022) | Play-Based Activities with a CoderBot Robot on a Pediatric Ward: A Case Study |  |  | * |
| Chen et al., (2023) | The Effects of Social Presence and Familiarity on Children-Robot Interactions |  |  | * |
| Cheng et al., (2021) | The effect of digital health technologies on managing symptoms across pediatric cancer continuum: A systematic review | * |  |  |
| Dosso et al., (2023) | Social robotics for children: an investigation of manufacturers' claims |  |  | * |
| Farrier et al., (2020) | Children's Fear and Pain During Medical Procedures: A Quality Improvement Study With a Humanoid Robot |  |  | * |
| Ferrari et al., (2023) | Design of Child-robot Interactions for Comfort and Distraction from Post-operative Pain and Distress |  |  | * |
| Foster et al., (2020) | Using AI-Enhanced Social Robots to Improve Children’s Healthcare Experiences |  |  | * |
| Foster et al., (2023) | Co-design of a Social Robot for Distraction in the Paediatric Emergency Department |  |  | * |
| Granberg et al., (2023) | And then there was one … incision. First single-port pediatric robotic case series |  |  | * |
| Jeong et al., (2017) | Huggable: Impact of embodiment on promoting verbal and physical engagement for young pediatric inpatients |  |  | * |
| Jeong et al., (2018) | Huggable: The impact of embodiment on promoting socio-emotional interactions for young pediatric inpatients |  |  | * |
| Jeong, Dos Santos, et al., (2015) | Designing a socially assistive robot for pediatric care |  |  | * |
| Jeong, Logan, et al., (2015) | A Social Robot to Mitigate Stress, Anxiety, and Pain in Hospital Pediatric Care |  |  | * |
| Kabacińska et al., (2025) | Social Robot Interactions in a Pediatric Hospital Setting: Perspectives of Children, Parents, and Healthcare Providers |  |  | * |
| Kałwa et al., (2024) | Can AI-assisted therapy (PARO robot) be a treatment option for children hospitalised in an acute mental health ward? |  |  | * |
| Kasimoglu et al., (2020) | Robotic approach to the reduction of dental anxiety in children |  |  | * |
| Lapedriza et al., (2024) | Deploying a Robotic Ride-on Car in the Hospital to Reduce the Stress of Pediatric Patients before Surgery |  |  | * |
| Larriba et al., (2016) | Externalising moods and psychological states in a cloud based system to enhance a pet-robot and child's interaction |  |  | * |
| Lau et al., (2020) | Humanoid robot-assisted interventions among children with diabetes: A systematic scoping review | * |  |  |
| Lee et al., (2019) | Efficacy of MEDi® preparation to manage children's pain and fear during iv inductions: A randomized-controlled trial |  | * |  |
| Lindsay et al., (2024) | A Socially Assistive Robot using Automated Planning in a Paediatric Clinical Setting |  |  | * |
| Littler et al., (2021) | Reducing negative emotions in children using social robots: Systematic review | * |  |  |
| Lu et al., (2011) | mediRobbi: An interactive companion for pediatric patients during hospital visit |  |  | * |
| Makino et al., (2022) | The robot assisted therapy using “aibo.” |  |  | * |
| Manaloor, Ali, et al., (2019) | Humanoid robot-based distraction to reduce pain and distress during venipuncture in the pediatric emergency department: a randomized controlled trial |  | * |  |
| Manaloor, Ma, et al., (2019) | LO63: Humanoid robot-based distraction to reduce pain and distress during venipuncture in the pediatric emergency department: A randomized controlled trial |  |  | * |
| Meghdari et al., (2016) | Conceptual design of a social robot for pediatric hospitals |  |  | * |
| Miller et al., (2019) | Feasibility and acceptability of an animatronic duck intervention for promoting adaptation to the in-patient setting among pediatric patients receiving treatment for cancer |  |  | * |
| Moerman & Jansens, 2021) | Using social robot PLEO to enhance the well-being of hospitalised children |  |  | * |
| Moerman et al., (2019) | Social robots to support children's well-being under medical treatment: A systematic state-of-the-art review | * |  |  |
| Nakadoi, 2015) | Usefulness of animal type robot in the treatment in child and adolescent psychiatric ward |  |  | * |
| Namlisesli et al., (2024) | The Effect of Use of Social Robot NAO on Children's Motivation and Emotional States in Special Education |  |  | * |
| Nguyen et al., (2024) | Innovating pediatric care with social robots to alleviate anxiety |  |  | * |
| Nichol et al., (2024) | Exploring the impact of socially assistive robots on health and wellbeing across the lifespan: An umbrella review and meta-analysis | * |  |  |
| O'Brien et al., (2021) | Exploring the design space of therapeutic robot companions for children |  |  | * |
| Øllgaard et al., (2025) | Utilizing a Social Robot as a Greeter at a Children’s Hospital |  |  | * |
| Or et al., (2025) | Effectiveness of social robots in improving psychological well-being of hospitalised children: A systematic review and meta-analysis | * |  |  |
| Palomaa et al., (2023) | Effectiveness of technology-based interventions compared with other non-pharmacological interventions for relieving procedural pain in hospitalized neonates: a systematic review | * |  |  |
| Pelizzari et al., (2025) | Integrating Robotics in Hospital and Home Education: A Systematic Review of Innovative Teaching Practices | * |  |  |
| Pourteimour & Kazemi, 2021) | The effectiveness of the robotic game kit on anxiety among hospitalized preschool children: A non-randomized controlled trial |  |  | * |
| Russell et al., (2021) | Use of a Social Robot in the Implementation of a Narrative Intervention for Young People with Cystic Fibrosis: A Feasibility Study |  |  | * |
| Signorelli et al., (2023) | A Review of Creative Play Interventions to Improve Children’s Hospital Experience and Wellbeing | * |  |  |
| Smakman et al., (2021) | Mitigating children’s pain and anxiety during blood draw using social robots |  |  | * |
| Son et al., (2022) | Play scenarios and interaction designs of a social robot for the development of children’s social-emotional intelligence by using the service design methodology |  |  | * |
| Stinson et al., (2016) | Using a humanoid robot to reduce procedural pain in children with cancer: a pilot randomized controlled trial |  |  | * |
| Sturgeon et al., (2017) | Robotic technology and palliative care education: the development of a “NAO robot” computer program |  |  | * |
| Tanaka et al., (2022) | Effects of artificial intelligence aibo intervention on alleviating distress and fear in children |  |  | * |
| Triantafyllidis et al., (2023) | Social robot interventions for child healthcare: A systematic review of the literature | * |  |  |
| Trost et al., (2019) | Socially assistive robots for helping pediatric distress and pain |  | * |  |
| Vallès-Peris et al., (2018) | Children's Imaginaries of Human-Robot Interaction in Healthcare |  |  | * |
| van Bindsbergen et al., (2022) | Interactive Education on Sleep Hygiene with a Social Robot at a Pediatric Oncology Outpatient Clinic: Feasibility, Experiences, and Preliminary Effectiveness |  |  | * |
| Weerarathna et al., (2023) | Human-Robot Collaboration for Healthcare: A Narrative Review | * |  |  |
| Williams et al., (2019) | Pilot Testing a Robot for Reducing Pain in Hospitalized Preterm Infants |  |  | * |
| Wright et al., (2025) | Robotic reading companions can mitigate oral reading anxiety in children |  |  | * |
| Zhang et al., (2022) | Understanding Design Preferences for Robots for Pain Management: A Co-Design Study |  |  | * |

**Table S4.** Summary of outcome and statistical results across included studies.

| Author (Year) | Outcome | Statistical results |
| --- | --- | --- |
| Alemi et al., (2016) | Anxiety, depression, anger | Anxiety (P=.002), depression (P=.019), anger (P=.012) |
| Ali et al. (2021) | Procedural pain and distress | Pain (NS^a^), distress (P=.047) |
| Beraldo et al. (2019) | State emotions | Anxiety (P=.047), fear (NS), sadness (NS), anger (NS), agitation (P=.029), confusion (P=.036), want to go home (P=.002), happiness (P=.039), want to play (NS), want to cuddle (P=.0036) |
| Chang (2023) | Procedural anxiety and emotional engagement | Anxiety (P<.05), emotional engagement (P<.05) |
| Franconi (2023) | Preoperative anxiety | Anxiety (P=.03) |
| Jibb (2018) | Procedural pain and distress | Pain (NS), distress (NS) |
| Lee-Krueger (2021) | Procedural pain and fear | Pain (NS), fear (NS) |
| Logan (2019) | State emotions and pain | Pain (parent reported: P<.01; child reported: NS), positive emotions (P<.05), negative emotions (NS) |
| Meghdari (2018) | Narrative Immersion | Emotion engagement (P<.03) |
| Okita (2013) | Pain and anxiety | Pain (P<.001), anxiety (P<.01) |
| Rossi (2021/2022) | salivary cortisol level and heart rate | Salivary cortisol level (P<.001), heart rate (P<.001) |
| Topçu (2023) | Postoperative anxiety and mobilization | Anxiety (P=.005), mobilization time (P=.042) |
| Trost (2020) | Procedural pain and fear | Pain (P=.758), fear (P=.47) |

^a^NS: nonsignificant

**Figure S1.** Funnel plot assessing potential publication bias for pain outcomes.

The plot shows slight asymmetry, suggesting possible small-study effects, although the limited number of studies (n = 5) restricts formal testing.

**Figure S2.** Funnel plot assessing potential publication bias for anxiety outcomes.

The plot appears relatively symmetrical, but the small number of studies (n = 3) limits interpretation and precludes formal testing.

**Figure S3.** Summary of the certainty of evidence assessment.

Certainty ratings were determined using the GRADE approach, considering five domains: risk of bias, inconsistency, indirectness, imprecision, and other considerations. All outcomes (pain, anxiety, fear, distress) were rated as having moderate certainty when compared with both standard and alternative care, with risk of bias being the primary reason for downgrading.


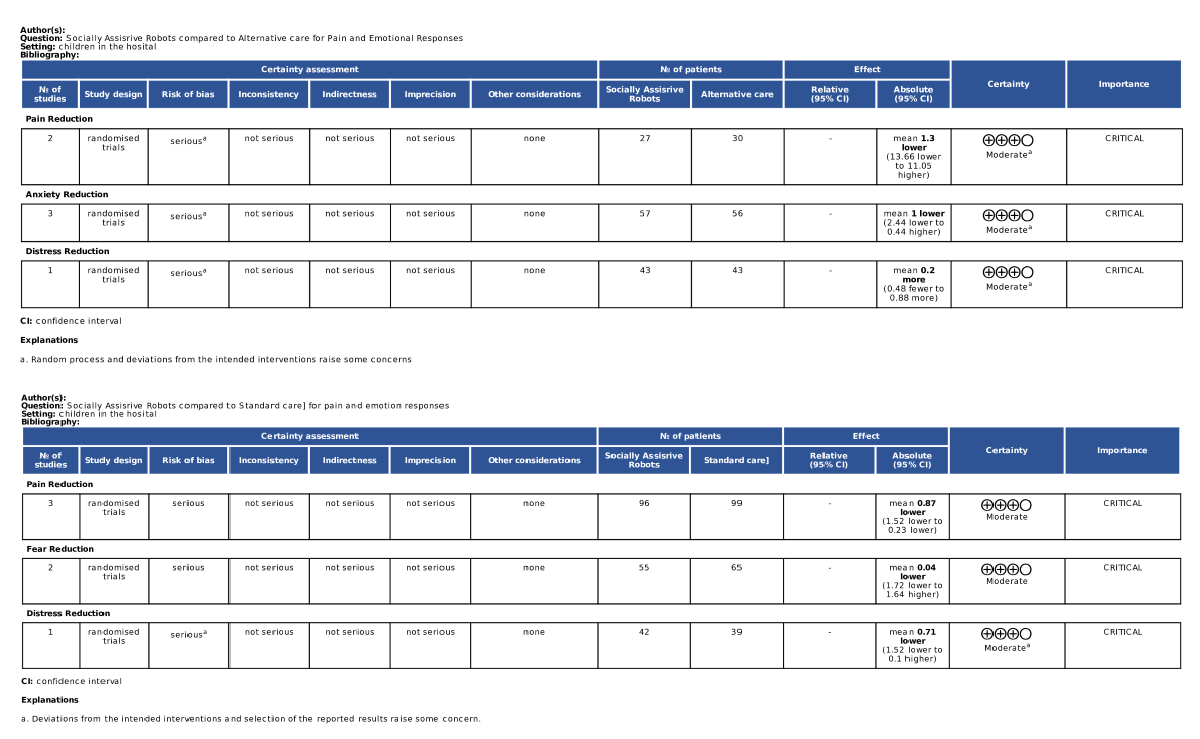

Supplement: Multimedia Appendix 2 [file jmir_v27i1e76427_app2.docx]
